# Complement C6 deficiency exacerbates pathophysiology after spinal cord injury

**DOI:** 10.1038/s41598-020-76441-3

**Published:** 2020-11-11

**Authors:** Diane Su, Mitra J. Hooshmand, Manuel D. Galvan, Rebecca A. Nishi, Brian J. Cummings, Aileen J. Anderson

**Affiliations:** 1 Department of Anatomy and Neurobiology, University of California, Irvine, Irvine, CA USA; 2 Institute for Memory Impairments and Neurological Disorders (iMIND), University of California, Irvine, Irvine, CA USA; 3 Sue and Bill Gross Stem Cell Research Center, University of California, Irvine, Irvine, CA USA; 4 Department of Physical Medicine and Rehabilitation, University of California, Irvine, CA USA

**Keywords:** Complement cascade, Spinal cord diseases

## Abstract

Historically, the membrane attack complex, composed of complement components C5b-9, has been connected to lytic cell death and implicated in secondary injury after a CNS insult. However, studies to date have utilized either non-littermate control rat models, or mouse models that lack significant C5b-9 activity. To investigate what role C5b-9 plays in spinal cord injury and recovery, we generated littermate PVG C6 wildtype and deficient rats and tested functional and histological recovery after moderate contusion injury using the Infinite Horizon Impactor. We compare the effect of C6 deficiency on recovery of locomotor function and histological injury parameters in PVG rats under two conditions: (1) animals maintained as separate C6 WT and C6-D homozygous colonies; and (2) establishment of a heterozygous colony to generate C6 WT and C6-D littermate controls. The results suggest that maintenance of separate homozygous colonies is inadequate for testing the effect of C6 deficiency on locomotor and histological recovery after SCI, and highlight the importance of using littermate controls in studies involving genetic manipulation of the complement cascade.

## Introduction

Traumatic spinal cord injury (SCI) causes a series of secondary degenerative events thought to exacerbate injury pathology^1–3^. One component of secondary injury after SCI is activation of the classical and alternative pathways of the complement cascade^1,4^. The complement cascade is composed of more than 30 soluble and membrane-bound proteins. The classical and alternative pathways converge on the terminal complement pathway, leading to the assembly of a multimeric protein complex, C5b-9, for which C6 is a necessary component^5,6^.

Previous studies suggest a detrimental role for C6/C5b-9 and activation of the terminal complement pathway in Central Nervous System (CNS) injury and disease. C5b-9 formation is thought to impede functional recovery and exacerbate pathology after CNS trauma^7–12^. CNS cells, particularly neurons and oligodendrocytes, are highly susceptible to C5b-9-mediated cell death in vitro^13–17^. In accordance with this observation, these cells express low levels of CD59, a membrane bound inhibitor of C5b-9 formation^18^, and deletion of CD59 increases C9 deposition in the injured spinal cord in association with increased histopathological inflammation, injury and demyelination compared to WT mice^11^. Moreover, C5b-9 is implicated in demyelination in experimental allergic encephalomyelitis (EAE)^8,19^, and demyelination-related Wallerian degeneration in peripheral nerve injury^10^. C5b-9 is also implicated as a mediator of tissue damage in atherosclerosis^20–22^, rheumatoid arthritis^23,24^, Alzheimer’s disease^25,26^, Huntington’s disease^27^, and Pick’s disease^28^.

In contrast, some evidence suggests that C5b-9 formation can result in not only cell lysis, but modulation of cell signaling when formed at sub-lytic levels. Sublytic C5b-9 induces polymorphonuclear leukocytes to produce reactive oxygen metabolites^29^ and increase endothelial synthesis of IL-8 and MCP-1^30^. In nervous system cells, sublytic C5b-9 induces Schwann cell proliferation^31,32^, stimulates oligodendrocyte proliferation, and enhances oligodendrocyte survival^33–37^. Taken together, these data suggest divergent or multiple roles for C6 and C5b-9 assembly in inflammation and CNS injury.

To investigate the role of C6 in SCI, we initially acquired Piebald-Viral Glaxo (PVG) C6-deficient (C6-D) rats from the University of Wales and PVG WT rats from Harlan laboratories. PVG C6-D rats carry a spontaneous mutation resulting in a lack of the C6 protein and are incapable of C5b-9 formation^2,9^. While heterozygous colony breeding to generate littermate controls for mouse transgenic studies is the standard for the field, this approach is not commonly applied for comparison of C6 deficiency in PVG rats. A literature search identifying papers that employed C6 WT and C6-D rats identified 48 studies. 28 studies utilized the C6-D PVG strain for xenograft or in vitro research and 2 describe characterizations of the C6 deficiency. Focusing on the 18 studies that used the C6-D PVG strain for investigation of experimental injury or disease, none detail the use of a littermate paradigm under the experimental methods. While animal source/breeding paradigm cannot be definitively determined due to lack of detail in some (5), the majority (13) report maintenance of C6-D and WT rats as separate colonies, or that the C6D and WT rats employed were ordered from separate companies. A further breakdown of these 18 studies reveals studies in each of the following disease/trauma models: infection^38^, experimental autoimmune neuritis^39^, lung inflammation^40^, arthritis^41^ experimental diabetes^42^, seizure^43^, experimental SCI^10^, the EAE model^8^, nephropathy^44^, kidney damage^45^, renal disease^46^, renal microvascular injury^47^, experimental nephrotic syndrome^48^, and 5 studies on nephritis^49–53^. The results reported here compare the effect of C6 deficiency on recovery of locomotor function and histological injury parameters in PVG rats under two conditions: (1) animals maintained as separate C6 WT and C6-D homozygous colonies; and (2) establishment of a heterozygous colony to generate C6 WT and C6-D littermate controls.

## Results

### Confirmation of C6-deficiency

**Figure 1:**
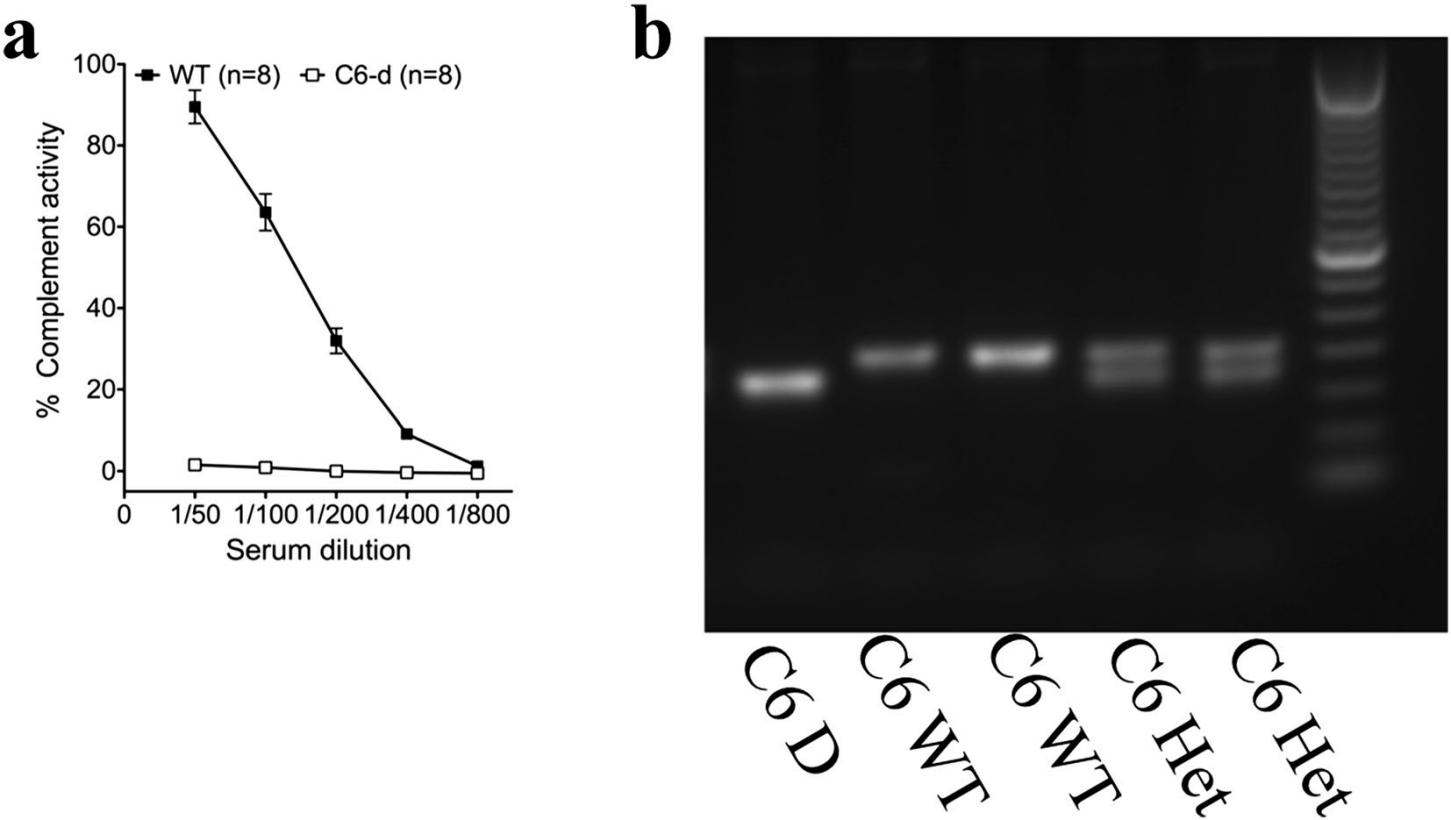
Genetic confirmation of C6 deficiency and hemolytic analyses of total complement activity in serum. (**a**) PVG H-C6-D rats showed complete loss of terminal complement activity compared to H-PVG WT (n = 8/group). (**b**) Genotypic results of PVG females showing genotypes F2-C6-D: 150 bp, F2–C6 WT: 200 bp, F2-C6 Het: 200 bp and 150 bp. Data points represent group means ± SEM. The original gel is provided in Supplementary Information S1.

**Figure 2:**
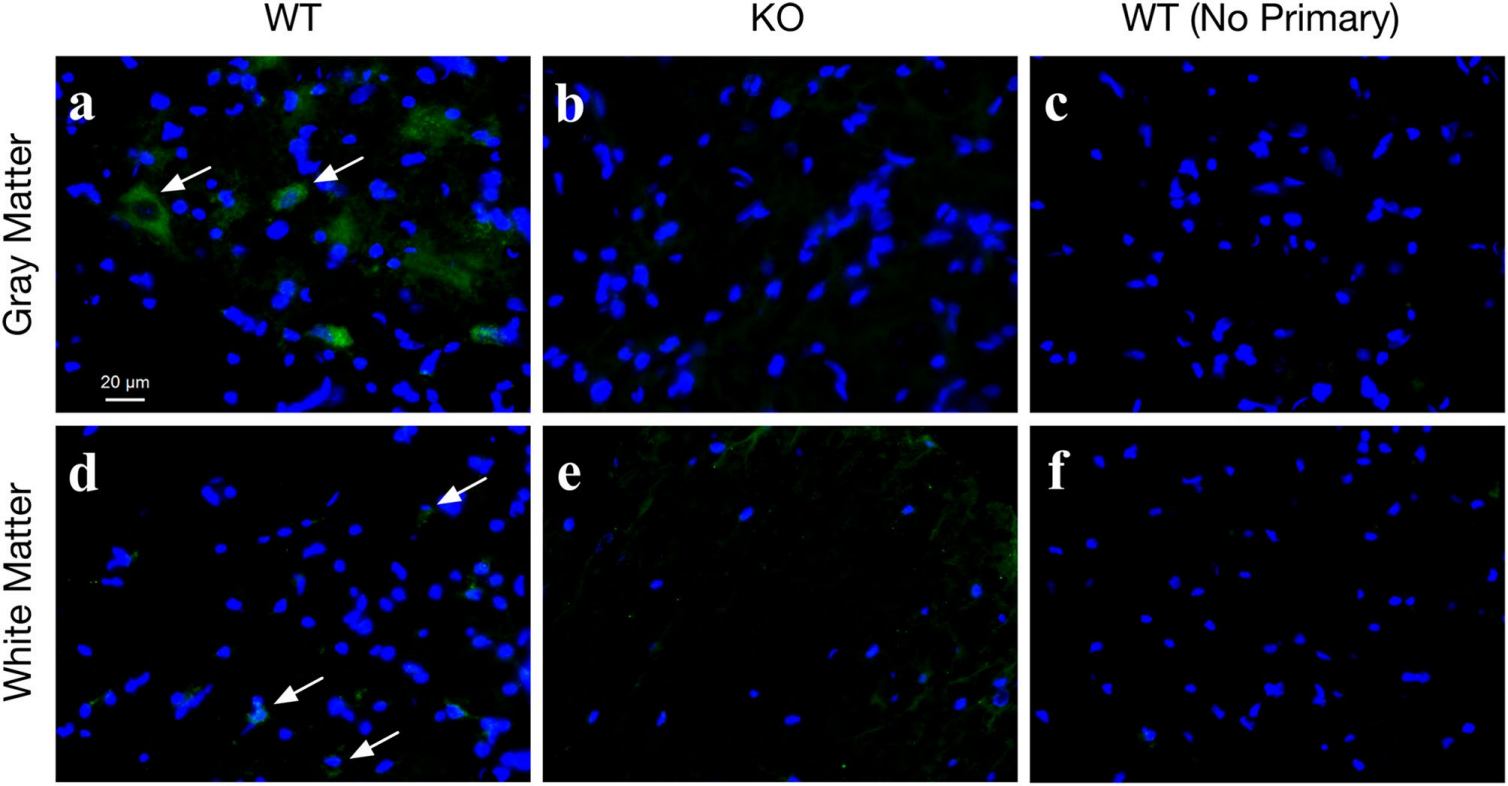
Presence of C5b-9 in the spinal cord following spinal cord injury in F2-C6 WT PVG (**a**,**d**) but not F2-C6-D (**b**,**e**) rats. (**a**) Representative images of gray matter and (**d**) white matter with C5b-9 staining (white arrows) in an injured F2-C6-WT PVG rat. (**b**) Representative images of gray matter and (**e**) white matter in an injured F2-C6-D. (**c**,**f**) Representative images of injured F2-C6-WT PVG gray matter and (**f**) white matter with no primary antibody. Scale bar 20 μm.

A PCR genotyping protocol was established for C6 deficiency by blast identification of a novel restriction site susceptible to Ava I cleavage and development of primer sequences enabling identification of restriction fragment lengths that could distinguish WT, heterozygous, and C6-D genotypes, as described under methods. In this protocol, WT (C6^+/+^) DNA is not cut and runs as a single band of ~ 200 bp, heterozygous (C6^+/−^) DNA exhibits incomplete digestion with two bands at  ~ 150, and 200 bp, and homozygous C6D (C6^−/−^) DNA exhibits complete digestion with one band at ~ 150 bp (Fig. 1b). The genotyping protocol was validated using rats sourced from homozygous and heterozygous breeding colonies, and by comparison with CH50 assay for dose-dependent lysis of IgG sensitized sheep erythrocytes (Fig. 1a). To further confirm that C5b-9 was present only in F2-C6-WT animals and not in F2-C6-D animals immunohistochemistry using an anti-C5b-9 antibody was conducted^54,55^. C5b-9 was observed only in the gray and white matter of F2-C6-WT SCI animals, and not in either F2-C6-D animals or sections for which the primary was omitted (Fig. 2).

### C6 deficiency impairs locomotor function in F2-C6 PVG rats but not H-C6 PVG rats

**Figure 3:**
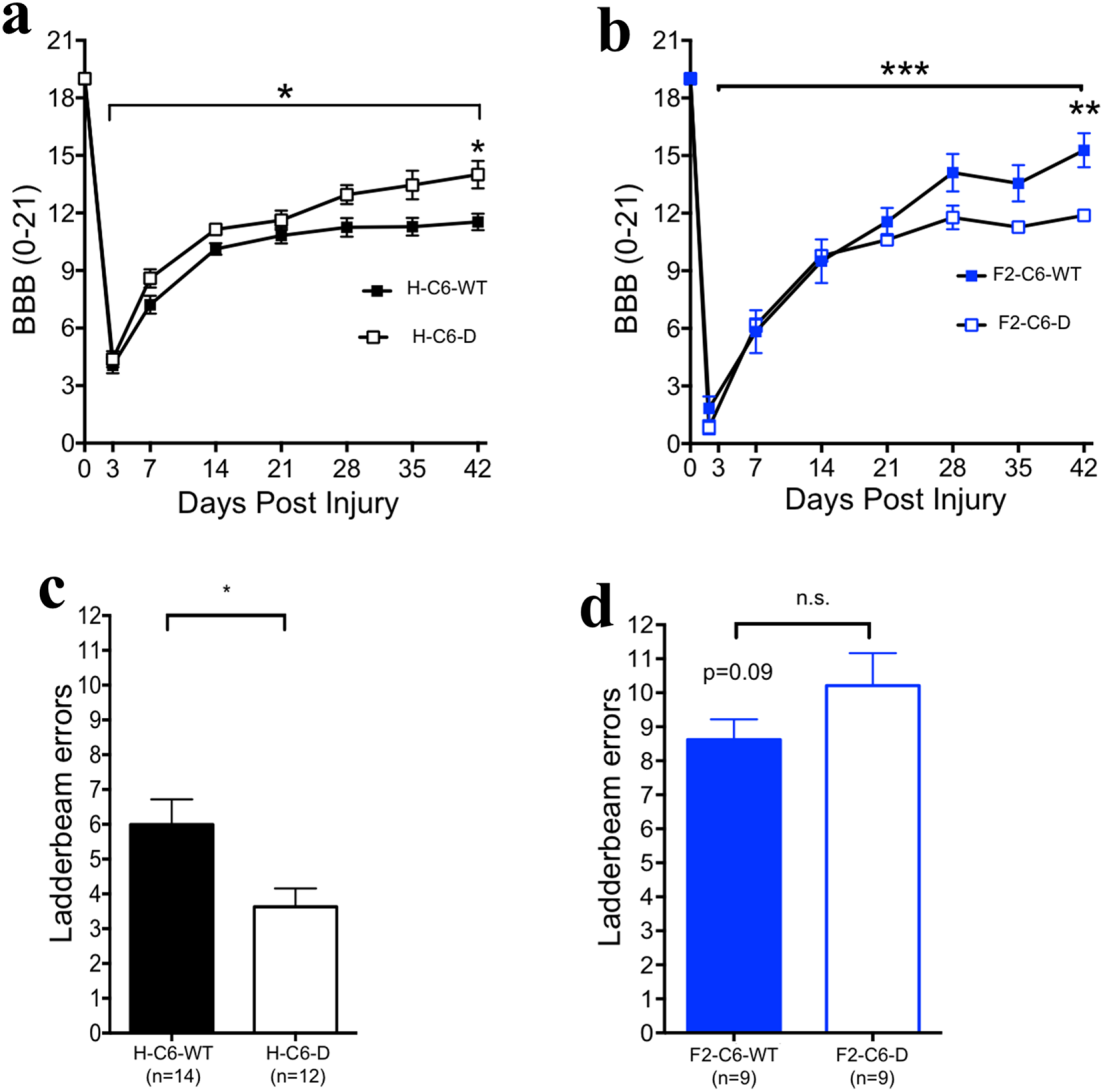
F2-C6-D PVG rats demonstrated reduced locomotor recovery compared to F2-C6 WT PVG rats. (**a**) Comparison of BBB scores showed that H-C6-deficient rats exhibited improved functional recovery throughout the duration of the study compared to H-C6 WT PVG rats (two way repeated measures ANOVA, *p < 0.05). Post hoc tests revealed a significant group difference 42 days post injury (Bonferroni’s multiple comparisons, *p < 0.05). (**b**) Comparison of BBB scores showed that F2-C6-deficient rats exhibited impaired functional recovery throughout the duration of the study compared to F2-C6 WT PVG rats (two way repeated measures ANOVA, *p = 0.0006). Post hoc tests revealed a significant group difference 42 days post injury (Bonferroni’s multiple comparisons post-hoc at day 42, **p < 0.01). (**c**) Terminal behavioral assessment (42 days post injury) on the horizontal ladderbeam task demonstrated that H-C6-D rats made significantly fewer errors compared to H-C6 WT (Student’s t test, #p < 0.05). (**d**) When comparing F2-C6-D rats performed no different from F2-C6 WT in ladderbeam errors. However there was a strong trend for F2-C6-D rats having more errors (Student’s t test, p = 0.09). Data points represent group means ± SEM.

For our initial experiment, rats were maintained as separate homozygous colonies as previously reported by many investigators (H-WT or H-C6-D PVG; homozygous colony cohort). In a subsequent experiment, we generated experimental animals from F2 crosses to yield littermate controls (F2-WT and F2-C6-D PVG; littermate control cohort). Animals from both cohorts received SCI at 4–6 months of age and were within 10% variation in weight (range 169.2–195.8 g). Multiple tasks were used to assess functional locomotor recovery after SCI. BBB is an open-field locomotor test that assesses gross functional improvements such as stepping and coordination. The higher a BBB score, the more functional the animal. Analysis in the homozygous colony cohort showed that on average, H-C6-D rats exhibited improved outcome vs. H-WT rats, as seen by higher BBB scores, suggesting that C6 deficiency improved locomotor outcome (Fig. 3a; WT: filled black squares, H-C6-D: open black squares; two way ANOVA p < 0.05, Bonferroni’s multiple comparisons Day 42 < 0.05). In contrast, this result was reversed when littermate controls were used: F2-C6-D rats exhibited impaired locomotor outcome, measured by lower BBB scores, vs. F2-WT rats (Fig. 3b; WT: filled blue squares; F2-C6-D open blue squares; two way ANOVA Genotype p = 0.0006, Bonferroni’s multiple comparisons Day 42 post-hoc Day 42 p < 0.01). Horizontal ladder beam analysis was consistent with these BBB results. Ladderbeam is a quantitative measure that assesses the number of errors made by the animal as it traverses a 20-rung horizontal ladder. Thus, the higher the number of errors, the less functional the animal. In the homozygous strain cohort, H-C6-D animals exhibited a decrease in average ladder beam errors vs. H-WT animals (Fig. 3c; WT: filled black bars, H-C6-D: open black bars; Student’s t test p < 0.05), consistent with improved recovery of function. Conversely, in the littermate control cohort, F2-C6-D rats showed no improvement, and a strong trend for an increase in errors vs. F2-WT rats (Fig. 3d; WT: filled blue bars; F2-C6-D open blue bars; Student’s t test p = 0.09).

Variation in behavioral recovery kinetics and plateau was observed across these sequential studies, with WT littermate control cohort animals performing significantly better than WT homozygous colony animals in BBB and worse in horizontal ladder beam testing. While this could suggest that homozygous colony and littermate control WT rats responded differently to SCI, these cohorts were injured more than 2 years apart, and could therefore have been affected by variables that cannot be excluded. For this reason, a conservative data analysis approach was taken, and no statistical comparisons between cohorts are made in these figures.

**Figure 4:**
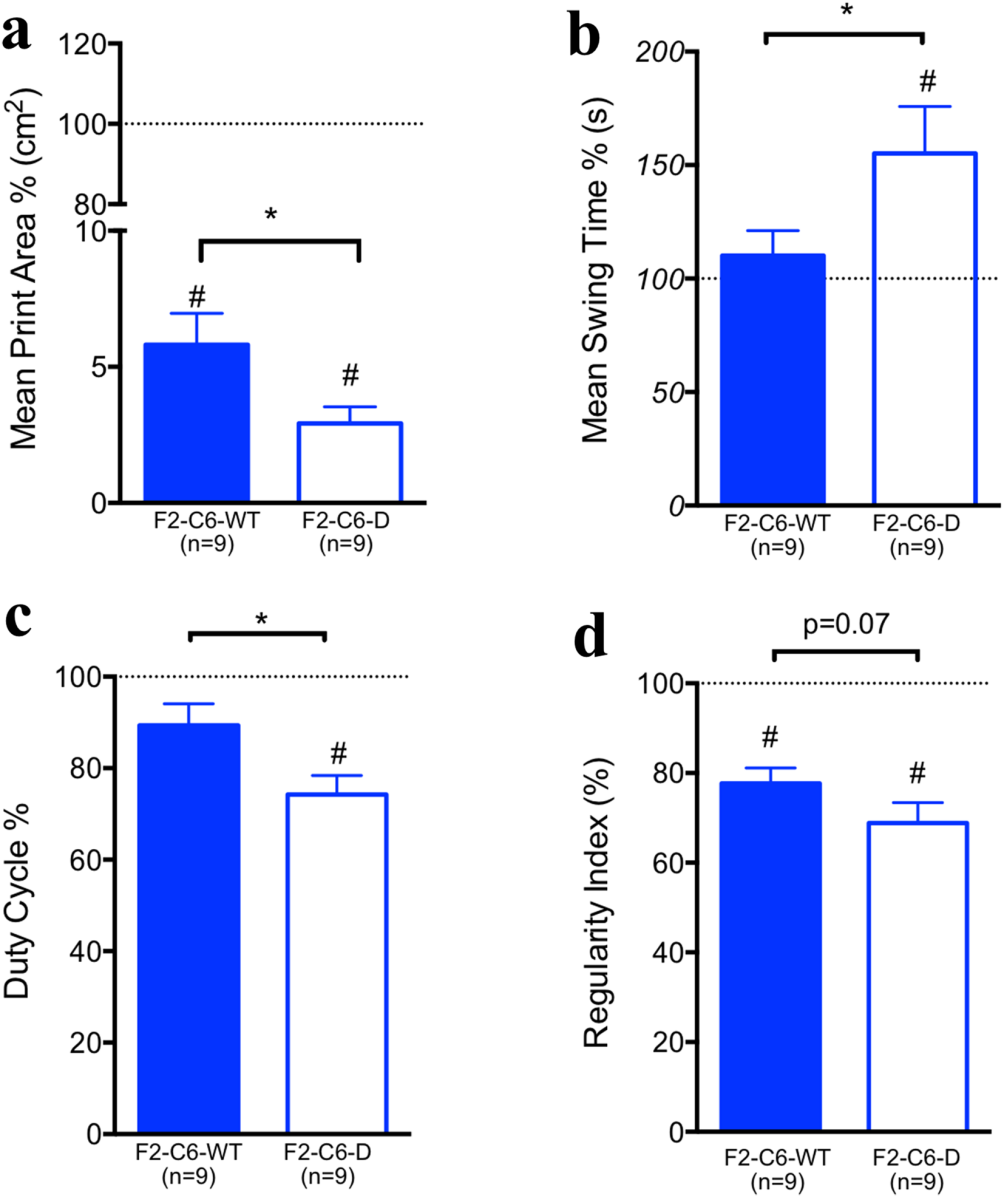
C6-deficiency in F2-C6-D animals impairs functional recovery as assessed by Catwalk. All animals were normalized to pre-injury baseline levels. A return to baseline (dotted line) suggests recovery to pre-injury levels. At 42 dpi F2-C6 deficient rats did not recover to baseline with respect to (**a**) mean print area, (**b**) mean swing time, (**c**) duty cycle, (**d**) regularity index. F2-C6 WT rats recovered to baseline levels with respect to all parameters except (**c**) mean print area and (**d**) regularity index (one sample t test, #p < 0.05). A significant change was observed between F2-C6 deficient and F2-C6 WT rats with respect to (**a**) mean print area (Student’s t test, p = 0.02), (**b**) swing time (p = 0.04), and (**c**) duty cycle (Student’s *t *test, * p = 0.03). There was also a post-injury trend towards a decrease in (**d**) regularity index score, indicative for coordination, for F2-C6-D rats compared to F2-C6 WT rats. Collectively, these data suggest that F2-C6-D rats, when normalized to baseline levels, and compared to F2-C6 WT rats, have decreased weight bearing ability on affected paws and decreased gait coordination. Data points represent group means ± SEM.

Horizontal ladder beam is most sensitive to recovery of function after SCI in the range of stepping recovery^56^. Because of the high performance plateau in the littermate cohort, we employed a supplemental assessment, Catwalk kinematic analysis, to assess fine parameters of gait after SCI in addition to stepping function (Fig. 4). Data were normalized to pre-injury data (dashed lines). One-sample t tests were used to compare baseline (dashed line) and post-injury performance; the closer the group mean is to baseline the better recovery post-SCI. Using this analysis, F2-C6-D rats consistently exhibited group means that showed decrements in recovery in comparison with WT animals. Both F2-C6 WT and F2-C6-D rats exhibited a significant decrease in print area, indicative of weight bearing, the higher the number, the more weight bearing, from pre-injury baseline (Fig. 4a, one sample t test ^#^p < 0.05), suggesting less weight support; however, F2-C6-D rats exhibited a significant further decrement in comparison with F2-C6-WT (Fig. 4a, Student’s t test *p = 0.02). A second parameter analyzed was mean swing time (the time interval between each consecutive placement of the same paw), for which a higher number indicates a deficit. While F2-C6 WT rats showed no difference in mean swing time vs. pre-injury baseline (Fig. 4b, one sample t test p > 0.05), F2-C6-D rats exhibited an increase in comparison with both pre-injury baseline (Fig. 4b, one sample t test ^#^p < 0.05) and F2-C6-WT animals (Student’s t test *p = 0.04). These data indicate a slowed step cycle and decrement in recovery compared to F2-C6 WT. A third parameter analyzed was duty cycle (the percentage of time spent standing within a step cycle), which is indicative of weight bearing. F2-C6-WT rats did not exhibit a significant decrease from pre-injury baseline for duty cycle, but F2-C6-D rats again demonstrated a significant decrement in comparison with both pre-injury baseline (Fig. 4c, one sample t test ^#^p < 0.05), and F2-C6 WT rats (Fig. 4c, Student’s t test *p = 0.03). The fourth parameter analyzed was regularity index (a measure of coordination). Both F2-C6-WT and F2-C6-D rats were significantly impaired vs. pre-injury baseline levels for regularity index (Fig. 4d, one sample t test ^#^p < 0.05); while not reaching significance, a strong trend for a further decrease in F2-C6-D rat was observed in comparison with F2-C6 WT rats (Fig. 4d, Student’s t test p = 0.08).

### C6 deficiency alters white matter sparing and lesion volume in homozygous strain H-C6 PVG but not in littermate control F2-C6 PVG rats

**Figure 5:**
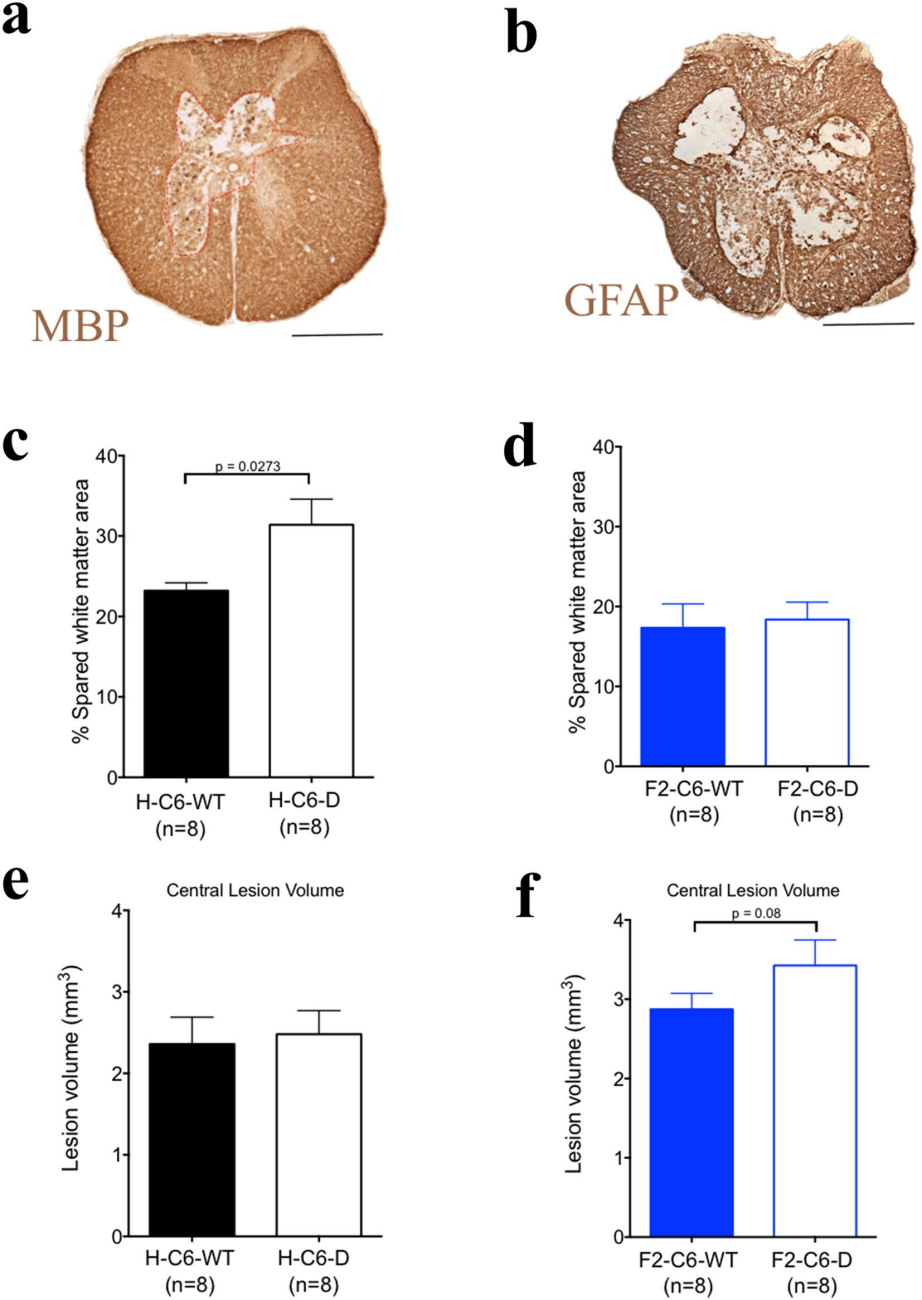
Unbiased stereological quantification of F2-C6-D PVG rats at 42 days. (**a**) Representative image of myelin basic protein staining near epicenter of injury in an F2-C6-D rat. (**b**) Representative image of GFAP staining near epicenter of injury in an F2-C6-D rat. (**c**) Stereological assessment demonstrated a significant increase in % spared tissue in H-C6-D rats (Student’s t test, *p < 0.05). (**d**) However, in F2-C6-D rats showed no difference in % spared tissue (Student’s t test, p = 0.4). (**e**) No significant differences were measured in central lesion volume in H-C6-D rats (Student’s t test, p > 0.05). (**f**) A strong trend for increased central lesion volume in F2-C6-D rats (Student’s t test, *p = 0.08). Data points represent group means ± SEM. Scale bar 500 μm.

We assessed whether C6 deficiency affected percent white matter sparing (Fig. 5a) or lesion volume (Fig. 5b) within the homozygous colony and littermate control cohorts using unbiased stereology. As noted above, statistical comparisons between cohorts should be approached with caution, because these cohorts were run at different times; however, no statistical differences were observed between H-WT and F2-WT rats for either SCI-induced lesion volume (H-WT mean 2.360 ± 0.3300 SEM N = 8), F2-WT 2.873 ± 0.2020 SEM N = 8; p = 0.2061; 2-tailed t test) or percent spared white matter (H-WT mean 23.20 ± 0.980 SEM, F2 WT mean 18.38 ± 2.17 SEM; p = 0.0625; 2-tailed t test). H-C6-D rats showed an increase in percent spared white matter area at the injury epicenter vs. H-C6-WT rats (Fig. 5c). In contrast, this measurement was unchanged between F2-C6-D and F2-C6 WT rats (Fig. 5d, Student’s t test, p = 0.4). Parallel analysis of central lesion volume in the homozygous strain cohort found no difference between H-C6-D vs. H-C6-WT rats (Fig. 5e). However, again in contrast, animals in the littermate control cohort exhibited a strong trend towards increased lesion size in F2-C6-D vs. F2-C6-WT rats (Fig. 5f, Student’s t test, p = 0.08). Taken together these histological data demonstrate divergent responses in lesion pathogenesis as well as locomotor recovery between homozygous colony and littermate control cohorts.

## Discussion

Here, we have compared the recovery differences of rats that were either maintained as separate homozygous colonies or generated from F2 crosses to yield littermate controls. Within cohort comparisons demonstrate that H-C6-WT vs. H-C6-D rats and F2-C6-WT vs. F2-C6-D rats have different results for both recovery of function and lesion pathogenesis after SCI. These results highlight the importance of the use of littermate controls in studies involving genetic manipulation of the complement cascade. While future studies should include comparisons between the different cohorts within the same experiment to also test for baseline differences, the current data indicate that there are functional differences due to maintenance in separate homozygous colonies versus heterozygous colonies to generate littermate controls.

The standard protocol in the literature has been to utilize animals from separate homozygous colonies for comparisons of C6 deficiency. A literature search identifying papers utilizing C6 WT and C6-D rats as a component of the experimental work identified 18 studies, a majority of which reported obtaining WT and C6 deficient rats from separate sources, highlighting a need to review findings using this model. Because of identified differences in complement sufficiency and activation between species, the C6-D PVG rat has been an important model for the study of complement and inflammation. C57Bl/6 mice, the most common background for genetically manipulated mice, have dramatically reduced C5b-9-hemolytic capacity in comparison with rats^57,58^. Indeed, we have previously reported that male C57Bl/6 mice fail to exhibit hemolytic activity in CH50 assays after SCI in comparison with male BUB mice or Sprague Dawley rats^58^. Accordingly, while transgenic mice can give insight into innate immune mechanisms in inflammation and injury, mouse models may have gaps with regard to the initiation of downstream immune activation. Together, these points highlight the importance of conducting studies in a rat model in which bred-in genetic variation between control and knockout/deletion animals is addressed.

C6-D PVG rats have been previously characterized and reported to derive from a specific autosomal recessive spontaneous mutation^59,60^; in parallel, tissue antigens, serum levels of other complement proteins, and spleen cell proliferation of C6-D rats have been reported to be identical to that of WT rats sharing the PVG background. Finally, these animals also do not exhibit either evidence of increased vulnerability to infection within a controlled vivarium environment, or evidence of differences at the level of immunity based on mixed lymphocyte reaction and alloantibody testing. However, it should be noted that the data presented cannot exclude the possibility of potential variabilities in blood or other immune parameters at baseline between colonies. Additionally, strain differences in SCI lesion pathogenesis are well recognized^61–67^. It would thus not be surprising that non-littermate WT and C6-D rats maintained in separate homozygous colonies for generations have diverged, resulting in different functional strain characteristics. In addition to genetic drift with colony maintenance practices, there are a number of reasons why maintenance of animals in separate colonies could yield such a result. For example, obtaining animals from different sources or housing in separate locations may lead to differences within the gut microbiome. Villarino et al. observed that C57Bl/6 mice that were sourced from different vendors, when exposed to the malaria parasite, differed in their severity response^68^. Gut microbiota has also been implicated in response to experimental autoimmune encephalomyelitis^69^, SCI^70,71^, and other CNS injury such as TBI^72^. In these studies, however, rats for both C6 WT and C6-D were bred in the same vivarium and fed the same chow; accordingly, the more likely scenario is that the differences in behavior and histology between the homozygous C6 WT and C6-D rat colony and heterozygous rat colony are due to the accumulation of genetic differences.

The results reported here suggest a surprising positive role for C5b-9 after nervous system injury, and are in contrast to studies have evaluated complement deficiency or knockout in SCI mice that are not complement sufficient or not bred to establish littermate controls. A positive role for C5b-9 in recovery after SCI could be consistent with the established role of complement in debris clearance, which is critical for regeneration and plasticity in the CNS. Studies have suggested C5b-9 presence can amplify the complement cascade through a positive feedback loop with C1q^10,19^. Amplification increases the recruitment of macrophages by an increase in C3a and C5a, which would be predicted to promote clearance of myelin debris that could otherwise inhibit remyelination. The presence of myelin and other debris may also indirectly increase stress or toxicity to, or impair the function of, surviving cells, leading to increased cell death as well as delayed remyelination^73–78^, negatively impacting motor recovery.

In sum, these results suggest that maintenance of separate homozygous colonies is inadequate for testing the effect of C6 deficiency on locomotor and histological recovery after SCI, and that comparison of F2-WT and F2-C6-D PVG rats provides a more appropriate model for assessing the role of complement in CNS injury. Moreover, these data point to a previously unidentified finding, that C5b-9 formation can exert beneficial functions after SCI, and presumably other types on CNS injury and damage.

## Materials and methods

### Animal numbers and exclusion criteria

For experiments comparing PVG rats maintained as separate homozygous colonies, PVG wildtype rats were purchased from Harlan (Indianapolis, Indiana) and C6 Deficient (C6-D) rats on a Piebald-Viral Glaxo (PVG) background were generously provided by Paul Morgan (University of Wales College of Medicine, U.K.). These strains are abbreviated as homozygous wildtype (H-WT) or homozygous C6-D-PVG), respectively. From these homozygous litters, H-C6 WT (n = 14) and H-C6-D (n = 12) females were used to assess motor function after spinal cord injury. For experiments comparing PVG rats generated as littermate controls, PVG rats WT purchased from Harlan were crossed with the homozygous PVG C6-D rats from the University of Wales, to generate F1 heterozygote C6-D rats at the University of California, Irvine; heterozygous males and females were then crossed to produce F2 litters with wildtype (F2-WT), heterozygous, and C6-D PVG rats (F2-C6-D PVG). F2-WT (n = 11) and F2-C6-D (n = 10) females were used to assess motor function after spinal cord injury. Only females were used in this study to facilitate manual bladder expression after SCI.

3 (WT, n = 2 and C6-D, n = 1) animals were excluded from final analysis; all exclusions were based on Grubbs test identification of outliers in behavioral data and/or post-injury weight monitoring.

### Genotyping

WT and C6-D genotypes were determined using PCR of DNA samples extracted from tail clips (Qiagen DNeasy Tissue Kit) followed by restriction enzyme digest and agarose gel electrophoresis. PCR was conducted using Taq PCR Master Mix (Qiagen) for 38 cycles: 4 m 94 °C; 45 s 94 °C; 1 m 50 °C; 1 m 72 °C; 10 m 72 °C. Primer sequences were as follows: C6 forward: TGCAGTAGGAATGGGGCTAA; C6 reverse: GAGAAAAGAGGCATTCCCAGT. PCR products were purified (Qiagen QIAquick PCR purification kit, DNA eluted in 30 µL NEB buffer, and 15 µl of eluted DNA product digested with 1 µl Ava I (New England Biolabs) overnight at 37 °C. PCR product digests were loaded onto a 1.5% agarose gel for electrophoresis and genotype identification relative to 100 bp DNA ladder as shown in Fig. 1a.

### Injury model

All experiments were conducted in accordance in an AALAC accredited vivarium with the UCI Institutional Animal Care and Use Committee (IACUC) approval. Rats were anesthetized with isofluorane gas anesthesia. A laminectomy was performed at thoracic vertebrae 9 (T9) and given 200 kilo dyne (kd) contusion injuries at T9 using the Infinite Horizon Impactor (Precision Systems and Instrumentation, Lexington, KY). After the contusion injury, a small piece of gel-foam was placed over the laminectomy location and the muscles overlying the injury was sutured and skin was closed using metal wound clips. Post-operative care included twice daily manual bladder expression until animals regained voluntary micturition, as well as administration of antibiotics and buprenorphine, as described previously^79^.

### Behavioral assessments

All behavioral testing and scoring was performed by individuals blinded to the experimental group and genotype.

#### Open field locomotion

The Basso Beattie Bresnahan (BBB) scale was utilized to evaluate gross locomotor recovery in injured animals weekly starting from 7 days post-injury (dpi) up to 42 dpi^80^. The BBB scale goes from 0 to 21 with the higher the BBB score the more functional the animal.

#### Horizontal* ladderbeam*

The horizontal ladder beam task was used as a terminal supplemental quantitative task with higher the number of errors, the less functional the animal^56,81–85^. Animals were tested at the end of the study prior to sacrifice. Prior to testing and video acquisition, rats were trained in a 20–30 m acclimation session. The apparatus length was 94.5 cm with 20 rungs spaced 2.54 cm apart. During the testing session, rats were videotaped from below; quantification of hindlimb errors was scored by slow motion video playback. Hindlimb errors from three test trials per rat were averaged for subsequent statistical analysis.

#### CatWalk gait assessment

Catwalk XT (version 7.1 Noldus Information Technology) was used to assess fine details of gait changes and allowed for easy quantification of a large number of gait parameters while animals are recorded crossing a walkway. Prior to testing and video acquisition, rats were trained in an acclimation session. Animals were recorded crossing the catwalk in the dark and data from three test trial per rat were averaged for statistical analysis.

### Tissue collection, sectioning and immunohistochemistry

At time of sacrifice, animals were perfused with 4% phosphate-buffered saline (PBS), followed by 4% paraformaldehyde in PBS. The T6–T12 segment of the spinal cord was dissected by the dorsal root to provide an anatomically defined region for stereology. Cords were further post-fixed in 20% sucrose/4% paraformaldehyde in PBS overnight at 4 °C. Spinal cords were frozen in isopentane at − 65 °C and stored at − 80 °C. Serial 30 μm thick coronal cryostat sections were cut on a CryoJane tape-transfer system (Leica Microsystems Inc., Cuffalo Grove, IL) at − 21 °C and collected in sets of 12 for immunohistochemistry.

#### White matter sparing and lesion volume

Slides were processed for antigen retrieval using a Buffer A (pH 6) in the Retriever 2100 system (PickCell Laboratories, Amsterdam, The Netherlands) before immunohistochemical staining. To assess white matter sparing, slides (1/12 section sampling) were dehydrated through a series of ethanol solutions (50%, 1 min; 70%, 3 min; 95%, 3 min; 100%, 5 min) and then rehydrated back through the alcohol series with fresh ethanol starting at 100% to 50% prior to incubation with anti-myelin basic protein primary antibody overnight (EMD Millipore). Anti-MBP was used at 1:750 and visualized with diaminobenzidine (DAB, Vector Labs, Carpinteria, CA). To assess central lesion volume, slides (1/12 section sampling) were incubated with anti-GFAP overnight (Dako). Anti-GFAP was used at 1:10,000 and visualized with diaminobenzidine (DAB, Vector Labs, Carpinteria, CA).

#### C5b-9 formation

Slides were processed for antigen retrieval using a trypsin buffer before immunohistochemical staining. To assess C5b-9 formation, mouse monoclonal antibody to Human SC5b-9 (Quidel A239) was used at 1:100 and was visualized with Donkey anti-mouse Alexa-flour 488 Fab (Jackson Immunoresearch) at a 1:500 dilution. A Hoescht counter stain at a dilution of 1:1000 was used to visual nuclei. Images were taken in the ventral horn using the Keyence BZ-X810 and a 60 × objective.

### Stereology

Stereological analyses for MBP and GFAP volume were performed on coronal sections (40–44 sections per animal) at 1/12 section sampling (360 µm apart) using StereoInvestigator software (Microbrightfield, Inc) using a 20 × objective. The Cavalieri estimator was used to quantify lesion volume, spared white matter volume, and percent spared white matter area. Quantification was performed by placing a random counting grid (100 μm × 100 μm) and markers that fell within the region of interest were counted. This data was used in the analysis to determined empirically a coefficient of error value that was < 0.10 for examined tissues. All histological analyses were performed by individuals blinded to group and genotype.

### Statistics

Prior to statistical analysis was performed a Grubbs’ test was performed to identify potential outliers from both behavioral and histological groups (α = 0.05), removing no more than a single outlier (non-iterative). Comparisons between C6 WT and C6-D rats for BBB were performed using repeated measures 2-way ANOVA with Sidak post-hoc test (Prism; GraphPad, San Diego, CA, USA). Catwalk data was normalized to each individual animal’s pre-injury value and comparisons performed using one sample *t *test analysis. Histological volume comparisons were conducted by Student’s t test, and MBP distribution from the lesion epicenter was conducted using repeated measures 2-way ANOVA.

## Supplementary information

Supplementary Information.
