# Supplementary material for: Complement C6 deficiency exacerbates pathophysiology after spinal cord injury
Source: Sci Rep. 2020 Nov 11;10:19500. doi: 10.1038/s41598-020-76441-3 (PMC7659012; doi:10.1038/s41598-020-76441-3)

Supplementary Gel Image for

**Complement C6 Deficiency Exacerbates Pathophysiology After Spinal Cord Injury**

Diane Su^1+^ and Mitra J. Hooshmand^1,2,3+^, Manuel D. Galvan^1^, Rebecca Nishi^3^, Brian J. Cummings^1,2,3,4^, and Aileen J. Anderson^1,2,3,4^*

^1^ Department of Anatomy and Neurobiology, University of California, Irvine, Irvine, CA, USA

^2^ Institute for Memory Impairments and Neurological Disorders (iMIND), University of California, Irvine, Irvine, CA, USA

^3^ Sue and Bill Gross Stem Cell Research Center, University of California, Irvine, Irvine, CA, USA

^4^ Department of Physical Medicine and Rehabilitation, University of California, Irvine, CA, USA

[**^*^**aja@uci.edu](mailto:*aja@uci.edu)

Areas used in Figure 1a are highlighted by a white square and correspond to the image in the main paper.

Figure 1a


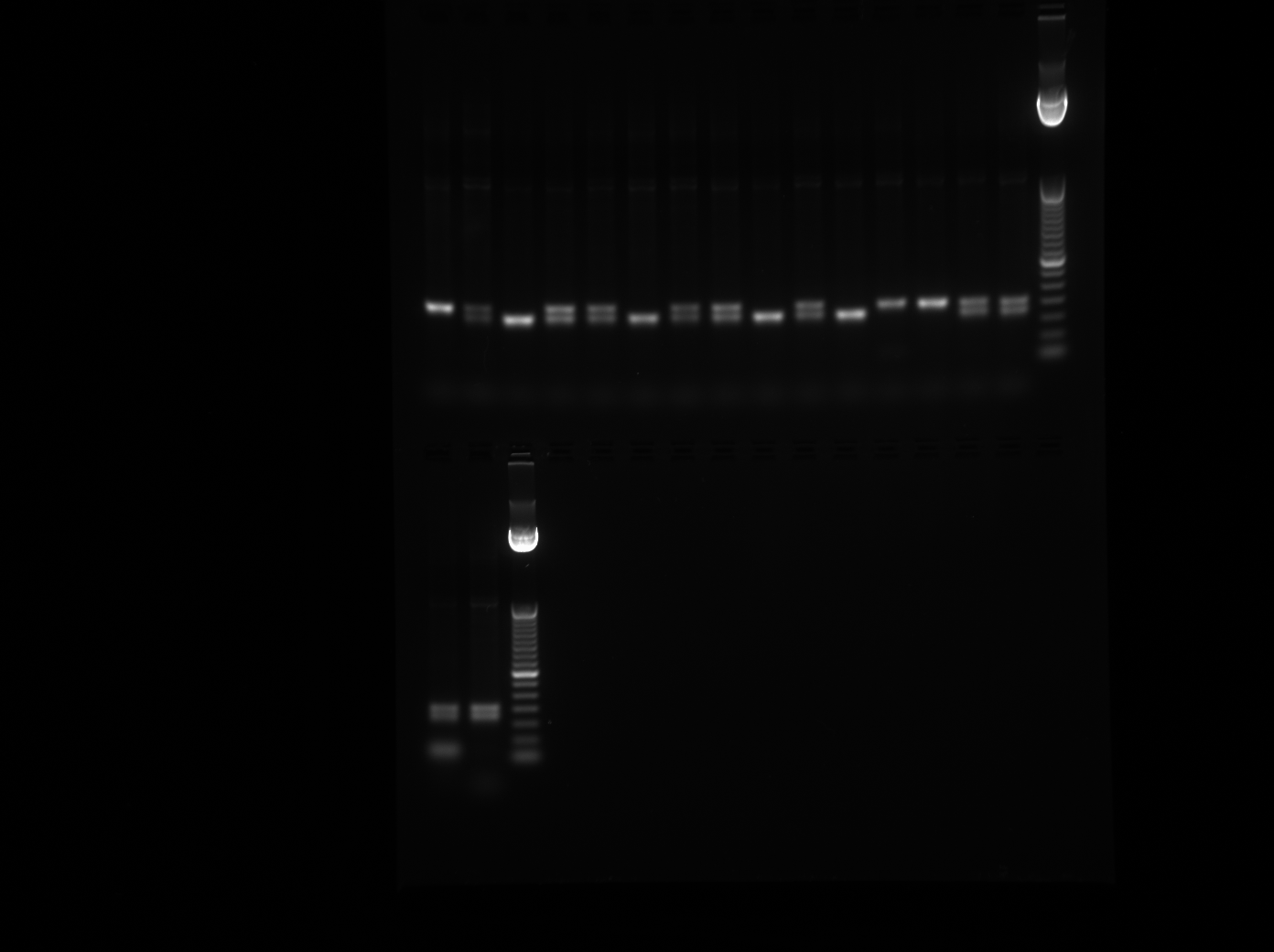

Supplement: Supplementary file 1 — Supplementary Information. [file 41598_2020_76441_MOESM1_ESM.docx]
